# Effect of Winter School Breaks on Influenza-like Illness, Argentina, 2005–2008

**DOI:** 10.3201/eid1906.120916

**Published:** 2013-06

**Authors:** Roberto C. Garza, Ricardo Basurto-Dávila, Ismael R. Ortega-Sanchez, Luis Oreste Carlino, Martin I. Meltzer, Rachel Albalak, Karina Balbuena, Pablo Orellano, Marc-Alain Widdowson, Francisco Averhoff

**Affiliations:** Centers for Disease Control and Prevention, Atlanta, Georgia, USA (R.C. Garza, I.R. Ortega-Sanchez, M.I. Meltzer, R. Albalak, M.-A. Widdowson, F. Averhoff);; Los Angeles County Department of Public Health, Los Angeles, California, USA (R. Basurto-Dávila);; Ministerio de Salud de la Nación, Buenos Aires, Argentina (L.O. Carlino, K. Balbuena, P. Orellano)

**Keywords:** Influenza, school closure, community mitigation, social isolation, Argentina, winter, viruses, respiratory infections

## Abstract

School closures are used to reduce seasonal and pandemic influenza transmission, yet evidence of their effectiveness is sparse. In Argentina, annual winter school breaks occur during the influenza season, providing an opportunity to study this intervention. We used 2005–2008 national weekly surveillance data of visits to a health care provider for influenza-like illness (ILI) from all provinces. Using Serfling-specified Poisson regressions and population-based census denominators, we developed incidence rate ratios (IRRs) for the 3 weeks before, 2 weeks during, and 3 weeks after the break. For persons 5–64 years of age, IRRs were <1 for at least 1 week after the break. Observed rates returned to expected by the third week after the break; overall decrease among persons of all ages was 14%. The largest decrease was among children 5–14 years of age during the week after the break (37% lower IRR). Among adults, effects were weaker and delayed. Two-week winter school breaks significantly decreased visits to a health care provider for ILI among school-aged children and nonelderly adults.

Children play a major role in the transmission of influenza within schools and households (*1*–*3*). These findings have garnered interest in use of school closures as critical nonpharmaceutical interventions during severe influenza epidemics to mitigate the spread of disease in the community (*4*). These closures might be especially useful in lower resource countries, where access to antiviral drugs and vaccines is relatively limited.

Recent studies have suggested that school closures might be effective for controlling the spread of influenza during a pandemic and reducing the spread of seasonal influenza (*5*–*11*). Results from modeling studies vary considerably; estimated case reductions because of school closures range from 40% to 90% (*8*,*11*). Studies that have relied on empirical analysis of disease data have reported narrower ranges, from 0 to 42% (*6*,*7*,*12*–*14*). For better understanding of the effectiveness of this mitigation strategy, additional studies that rely on multiple years of disease data from other influenza-related school closure experiences are needed.

Argentina, a middle-income country in the Southern Hemisphere, has annual winter school breaks in all provinces. We examined the weekly syndromic surveillance data for influenza-like illness (ILI) from Argentina and estimated the effectiveness of these breaks on incidence of ILI in the community.

## Methods

### Overview

For all provinces in Argentina, we used province-specific, age-stratified surveillance data on weekly reported hospitalizations and outpatient visits attributable to ILI during 2005–2008 to construct Poisson regression models. We then correlated these data with province-specific school calendars for the same periods. We compared the observed and expected rates of ILI cases during 3 periods: before, during, and after the 2-week winter school break.

### Data

Each week, each of the country’s 23 provinces and the city of Buenos Aires report all visits to a health care provider for ILI (hospitalizations and outpatient visits, hereafter referred to as ILI cases) from all Argentina government health care providers and facilities, including hospitals and clinics, to the Argentina Ministry of Health through its National System for Health Surveillance (Sistema Nacional de Vigilancia de la Salud; SNVS). According to the Argentina Census Bureau, 48.1% of the population has no form of health insurance. The SNVS captures health care visits made by these persons as well as by those who seek health care at government facilities (*15*).

Implementation of the SNVS began in 2000 and became fully functional nationwide in 2005. Weekly surveillance data are stratified in the following 10 age groups: <1 y, 1 y, 2–4 y, 5–9 y, 10–14 y, 15–24 y, 25–34 y, 35–44 y, 45–64 y, and ≥65 y. The case definition of ILI for SNVS reporting is temperature >100.4°F with cough or sore throat, possibly accompanied by weakness, muscle pain, nausea or vomiting, runny nose, conjunctivitis, inflammation of the lymph nodes, or diarrhea.

School calendars for each province, including the dates for winter school breaks for public primary and secondary schools, were obtained directly from the Argentina Ministry of Education. Each province independently determines its school calendar at the beginning of the school year; thus, the dates of winter school breaks vary across provinces and years and might not coincide with seasonal influenza peaks. This variation provides a natural experiment for our evaluation. We used data from Argentina’s 2001 population census (*16*) for province-level population estimates and assumed that populations remained constant over the study period.

### Analysis

To estimate the effect of winter school breaks on ILI cases, we fitted a statistical regression model to ILI surveillance data for each age group and then measured the difference between observed and expected incidence of ILI cases. For our statistical model, we used Poisson regressions with a Serfling specification, a sinusoidal equation that accounts for annual seasonal patterns in ILI outcomes (*17*). By incorporating annual seasonality of influenza activity into the regression model, we could estimate the effect of timing of winter breaks on ILI incidence while controlling for decreases and increases in ILI incidence associated with the annual seasonal patterns characteristic of influenza. The dependent variable was the number of ILI visits for each province, by age group and week. The independent variables were time trends (linear and quadratic effects), sinusoidal terms to account for seasonal patterns of ILI or influenza activity; fixed effects for geographic region, year, weeks with winter school breaks; and variables for each of the 3 weeks that immediately preceded or followed winter school breaks (Technical Appendix). We also included interaction terms between year and region, year and sinusoidal terms, and region and seasonal terms (Technical Appendix). Our model allowed for differences in the seasonality of ILI visits across regions and years, which might result from climate variety in Argentina, province response to ILI, and the timing and peak of influenza circulation in the local community. Model fit was evaluated by using pseudo R^2^ values and comparing plots of predicted versus reported ILI cases.

Effects of winter school breaks on ILI cases were estimated separately for each of the following age groups: <5 y, 5–14 y, 15–24 y, 25–44 y, 45–64 y, and ≥65 y. These age groups represent aggregate data from the SNVS that better match groups of persons at different school grades or different stages of life. We report the results of these estimations as ILI incidence rate ratios (IRRs) for the 3 weeks immediately before, 2 weeks during, and 3 weeks immediately after the winter school breaks. IRRs, as used in our analysis, estimate whether incidence of ILI-associated visits to a physician in a particular week were lower, higher, or did not deviate from the expected seasonal ILI patterns. That is, statistically significant IRRs <1 or >1 indicate that ILI cases during a particular week for a specific age group were below or above the estimated seasonal trend, respectively. Conversely, an IRR that is not statistically significant suggests that the number of ILI cases in a particular week did not deviate from expected cases of ILI. We repeated this analysis for each of Argentina’s 6 regions: Argentine Northwest, Gran Chaco, Mesopotamia, Cuyo, Pampas, and Patagonia.

We also estimated the number of ILI episodes prevented by winter school breaks, defining them as the difference between observed and expected ILI in a scenario without winter school breaks. That is, we assumed that there were no winter school breaks and used the results of the regression model to predict ILI cases without the reductions in ILI visits with the weeks during and immediately after school breaks.

The investigation protocol was reviewed by the Argentina Ministry of Health and the Centers for Disease Control and Prevention and was given a nonresearch determination. All analyses were performed by using Stata statistical software version 10.1 (StataCorpLP, College Station, TX, USA).

## Results

**Figure 1:**
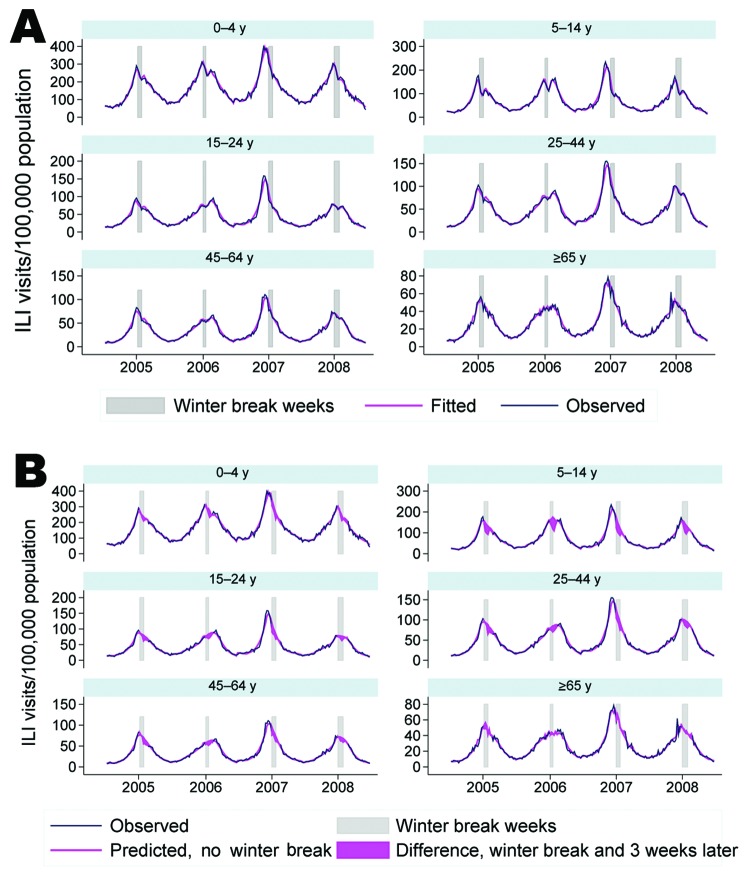
Observed and predicted cases of influenza-like illness (ILI), by age group, Argentina, 2005–2008. A) Observed and model-fitted predictions of incidence. B) Differences between observed cases and model predictions removing the estimated effect of winter school breaks.

During 2005–2008, a total of 4,376,181 cases of ILI were reported to the SNVS; an average of ≈20,900 cases occurred per week, or an average of 63 cases per 100,000 population nationwide (based on population estimates from the 2001 population census) (*16*). Rates of reported ILI varied significantly across provinces over the 4-year study period, from 11 cases per 100,000 population in La Rioja Province to 169 cases per 100,000 population in Misiones Province (Table 1). Reports of ILI cases followed a seasonal pattern; yearly peak activity occurred during the winter months of May–August (Figure 1, panel A). Most provinces did not consistently conduct their school breaks during the same week every year, and provinces that began the school breaks earlier in a particular year did not necessarily do so every year (Table 1).

**Table 1:** Incidence of visits to a health care provider for influenza-like illness and timing of first week of winter school break in Argentina, by province, 2005–2008

City or province	Region	2001 Argentina population, no. (%)	Average weekly incidence (95% CI)*	Epidemiologic weeks of winter break†			
				2005	2006	2007	2008
Buenos Aires City	Pampas	2,776,138 (7.7)	16 (0.63–75.64)	28–29	28–29	30–31	31–32
Province							
Buenos Aires	Pampas	13,827,203 (38.1)	43 (3.63–149.38)	28–29	29–30	30–31	31–32
Catamarca	Northwest	334,568 (0.9)	113 (14.47–299.56)	28–29	29–30	29–30	29–30
Cordoba	Pampas	3,066,801 (2.7)	56 (5.83–184.77)	28–29	29–30	28–29	28–29
Corrientes	Mesopotamia	930,991 (1.1)	59 (7.81–199.56)	28–29	29–30	28–29	29–30
Chaco	Gran Chaco	984,446 (8.5)	143 (36.75–401.55)	29–30	29–30	29–30	30–31
Chubut	Patagonia	413,237 (2.6)	100 (22.93–244.67)	28–29	29–30	28–29	28–29
Entre Rios	Mesopotamia	1,158,147 (3.2)	113 (19.09–341.92)	28–29	29–30	28–29	29–30
Formosa	Gran Chaco	486,559 (1.3)	122 (24.31–411.54)	28–29	29–30	28–29	29–30
Jujuy	Northwest	611,888 (1.7)	127 (33.03–325.24)	28–29	28–29	29–30	29–30
La Pampa	Pampas	299,294 (0.8)	84 (0–263.86)	28–29	28–29	28–29	29–30
La Rioja	Northwest	289,983 (0.8)	11 (0–38.81)	28–29	29–30	28–29	29–30
Mendoza	Cuyo	1,579,651 (4.4)	57 (0.35–171.09)	28–29	29–30	28–29	29–30
Misiones	Mesopotamia	965,522 (2.7)	169 (28.49–604.50)	29–30	29–30	28–29	29–30
Neuquen	Patagonia	474,155 (1.3)	78 (9.55–233.61)	29–30	29–30	29–30	29–30
Rio Negro	Patagonia	552,822 (1.5)	57 (10.40–149.10)	30–31	29–30	29–30	29–30
Salta	Northwest	1,079,051 (3.0)	158 (58.76–333.69)	28–29	29–30	28–29	29–30
San Juan	Cuyo	620,023 (1.7)	36 (2.66–107.17)	28–29	28–29	28–29	29–30
San Luis	Cuyo	367,933 (1.0)	75 (9.37–217.36)	28–29	28–29	28–29	29–30
Santa Cruz	Patagonia	196,958 (0.5)	58 (9.48–138.03)	29–31	28–29	28–29	29–30
Santa Fe	Pampas	3,000,701 (8.3)	38 (2.59–141.07)	28–29	29–30	28–29	29–30
Santiago del Estero	Gran Chaco	804,457 (2.2)	90 (15.83–278.47)	29–30	29–30	29–30	29–30
Tucuman	Northwest	1,338,523 (3.7)	102 (11.39–296.37)	28–29	29–30	28–29	29–30
Tierra del Fuego	Patagonia	101,079 (0.3)	83 (0–223.01)	29–30	29–30	29–30	29–30
All Argentina	Not applicable	36,260,130	63 (11.68–200.37)	Not applicable			

*Visits/100,000 population. †For reference, epidemiologic week 28 is typically in mid-July.

The regression models for each age group provided a good statistical fit to the data; pseudo R^2^ values ranged from 0.66 to 0.72. Model goodness-of-fit can also be observed when comparing predicted values against observed number of ILI cases (Figure 1, panel A).

**Figure 2:**
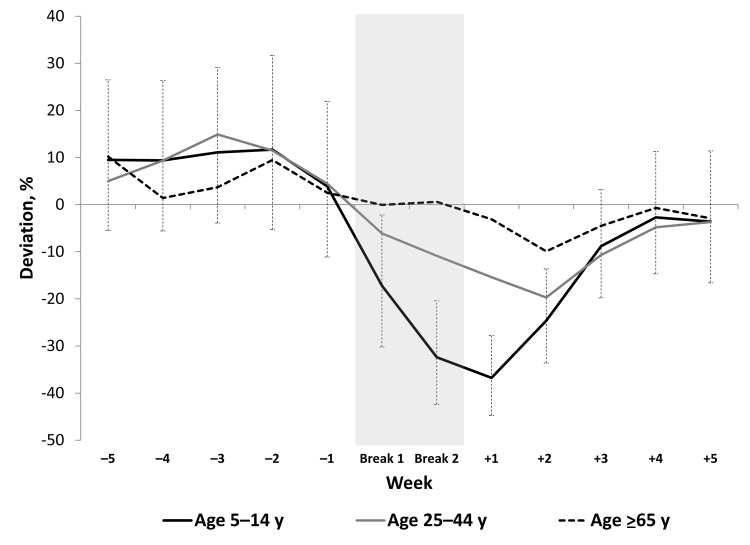
Estimated deviation from predicted incidence rates for influenza-like illness relative to winter break, by week and age group, Argentina, 2005–2008. Dashed lines show the 95% CI for the incidence rate ratios of age group 5–14 years because this is the age group of interest and because it simplifies the display of these results. Statistical significance for the other age groups is shown in Table 2.

Except for the age groups <5 and ≥65 years, lower incidence rates for ILI visits (i.e., IRR<1) were estimated for all groups for at least 1 of the weeks during or after winter school break, but this effect varied by age group in strength and timing relative to the start of the break (Table 2). Statistically significant IRRs for the age group 15–24 years occurred during the 2 weeks after the winter break. Among adults, 25–44 years of age, significant deviations from seasonal trends in ILI visits were observed for the second week (IRR = 0.83, p = 0.009) after the winter break. Among all age groups, incidence of ILI visits returned to regular seasonal patterns 3 weeks after the end of the winter break (Figure 2; Table 2).

**Table 2:** Estimated IRRs of visits to a health care provider for ILI surrounding winter school breaks, by patient age, Argentina, 2005–2008*

Patient age, y	Time in relation to winter school break, IRR (95% CI)								Pseudo R^2^
	3 wk before	2 wk before	1 w before	Wk 1 of break	Wk 2 of break	1 wk after	2 wk after	3 wk after	
0–4	1.09 (0.96–1.24)	1.09 (0.96–1.23)	1.10 (0.97–1.25)	1.01 (0.89–1.15)	0.95 (0.84–1.07)	0.89 (0.79–1.01)	0.93 (0.83–1.05)	1.03 (0.93–1.15)	0.70
5–14	1.09 (0.95–1.26)	1.10 (0.94–1.29)	1.03 (0.90–1.19)	**0.83** **(0.72–0.95)**	**0.69** **(0.61–0.79)**	**0.67** **(0.59–0.76)**	**0.81** **(0.71–0.92)**	0.96 (0.85–1.10)	0.70
15–24	1.15 (0.97–1.35)	1.10 (0.95–1.29)	1.04 (0.90–1.19)	0.91 (0.80–1.04)	0.88 (0.77– 1.01)	**0.87** **(0.77–0.99)**	**0.86** **(0.76–0.97)**	0.95 (0.85–1.08)	0.72
25–44	1.11 (0.94–1.32)	1.07 (0.92–1.26)	1.02 (0.87–1.19)	0.93 (0.80–1.08)	0.90 (0.77–1.05)	0.87 (0.75–1.00)	**0.83** **(0.72–0.95)**	0.93 (0.81–1.07)	0.72
45- 64	1.10 (0.94–1.30)	1.07 (0.91–1.26)	1.03 (0.87–1.21)	0.95 (0.82–1.11)	0.92 (0.78–1.08)	0.91 (0.78–1.06)	**0.86** **(0.74–0.99)**	0.91 (0.79–1.05)	0.72
>65	1.05 (0.85–1.31)	1.14 (0.93–1.41)	1.05 (0.87–1.27)	1.04 (0.84–1.28)	1.09 (0.90–1.31)	1.05 (0.87–1.27)	0.99 (0.84–1.17)	1.06 (0.88–1.27)	0.66

*IRRs were used to estimate whether incidence of ILI-associated visits in a particular week were lower, higher, or did not deviate from the expected seasonal ILI patterns. Each row represents a separate regression model. **Boldface** indicates statistically significant changes at the 0.05 confidence level. IRR, incidence rate ratio; ILI, influenza-like illness.

The largest decrease in observed ILI cases was among school-aged children (5–14 years of age). For this age group, ILI-associated health care visits were 33% (p<0.05) lower than expected during the 2 weeks of winter break and the 2 weeks after winter break; this decrease included a 17% decrease (i.e., 1–IRR, where IRR = 0.83, p = 0.008) in the first week of winter school break. The largest deviation from seasonal trends, 33% (IRR = 0.67, p<0.001), was observed during the first week after the school break (Table 2; Figure 2). This significant decrease in ILI among school-aged children 5–14 years of age was also found within each of the 6 regions in Argentina (Table 3).

**Table 3:** : Estimated reduction in number of influenza-like illness cases as a result of winter school breaks in Argentina, Argentina, 2005–2008*

Patient age, y	Region, no. cases prevented (% reduction; 95% CI)						
	Argentina	Pampas	Noroeste	Gran Chaco	Mesopotamia	Cuyo	Patagonia
0–4	7,044 (5; −4 to 15)	**9,875** **(18; 5 to 32)**	908 (5; −6 to 16)	1,380 (8; −4 to 19)	576 (2; −8 to 13)	141 (2; −20 to 24)	514 (12; −10 to 33)
5–14	**38,916** **(33; 19 to 47)**	**19,907** **(39; 19 to 59)**	**5,639** **(28; 12 to 44)**	**4,776** **(33; 15 to 51)**	**4,683** **(24; 9 to 39)**	**3,244** **(48; 19 to 77)**	**2,293** **(44; 19 to 69)**
15–24	**10,064** **(14; 1 to 26)**	**4,388** **(15; 2 to 29)**	**3,365** **(21; 7 to 36)**	**2,126** **(27; 10 to 43)**	1,395 (12; −1 to 26)	1,017 (18; −3 to 40)	**1,178** **(23; 6 to 39)**
25–44	**16,074** **(13; 0 to 26)**	**9,519** **(20; 6 to 34)**	**5,715** **(21; 9 to 33)**	**3,170** **(25; 10 to 40)**	1,235 (9; −4 to 21)	**2,239** **(23; 4 to 42)**	**1,236** **(13; 1 to 26)**
45–64	6,583 (10; −3 to 23)	**3,711** **(13; 1 to 26)**	**4,076** **(28; 15 to 41)**	**1,424** **(22; 5 to 39)**	518 (6; −7 to 20)	419 (8; −11 to 27)	335 (7; −6 to 21)
>65	−1,136 (−4; −19 to 11)	1,067 (9; −5 to 22)	263 (5; −10 to 20)	517 (18; −1 to 37)	−164 (−4; −18 to 9)	−136 (−8; −34 to 18)	54 (4; −20 to 27)
Total	77,545 (14)	48,467 (22)	19,966 (19)	13,393 (22)	8,243 (10)	6,924 (20)	5,610 (18)
*Except for the last row, each cell represents a separate regression model. **Boldface** indicates significant changes at the 0.05 confidence level. No confidence intervals are reported in the last row because those cells only represent the sum of other cells in each column.							

Assuming no winter school breaks, we estimated that during 2005–2008, without school breaks there would have been 77,290 more ILI cases, a 14% increase over observed cases during that period (Table 3; Figure 1, panel B). More than half (38,859 [50.3%]) of the difference in ILI cases occurred among children 5–14 years of age; the group that experienced the second largest difference were young adults, 25–44 years of age (15,989 [20.7%]).

## Discussion

Our analysis of weekly rates of ILI cases reported by health care providers throughout Argentina for 2005–2008 found that winter school breaks were associated with significant decreases in the number of cases in school-aged children and in the community at large. The effect on ILI followed a stepwise trend; the 5–14 year age group experienced the initial decrease in ILI during the first week of winter school break, lasting 4 weeks (which includes the first 2 weeks back in school). The effect was then seen among other age groups, each experiencing a smaller decrease in ILI. These findings are significant and biologically and epidemiologically plausible because the effect of school closures on disease transmission could be expected to begin with students and subsequently move to parents of these students and eventually to older family members. These results are consistent within each of Argentina’s 6 regions and across the country as a whole.

Our findings support those of previous studies, suggesting that school closure can be an effective mitigation strategy for limiting the spread of pandemic influenza (*5*–*7*,*9*–*11*,*18*). These findings are comparable to those of Cauchemez et al., who also studied ILI surveillance data from outpatient visits in France and found a 16%–21% decrease in seasonal influenza cases that were attributed to winter breaks in that country (*6*). Our results are also consistent with a study that found a 42% decrease in diagnoses of respiratory infections and a 28% decrease in visits to physicians during a 2-week period of school closure in Israel (*14*).

As an ecologic study that uses a time-trend design, our study is subject to the limitation that our aggregate data cannot be used to make inferences on causality or the effect on individual persons (*19*). Our findings represent reductions in ILI-associated visits to a health care provider, and not laboratory confirmed influenza, although the seasonal increase in ILI among older children and adults is strongly associated with influenza circulation (*20*). Furthermore, the surveillance data we analyzed were obtained primarily from public hospitals and clinics, which account for ≈43% of the health clinics in Argentina and thus might not be representative of the community (*21*). Data are not fully representative, mainly because only 7% of the data reported to the SNVS come from private hospitals, clinics, and providers (*22*). Moreover, our data only included dates of winter breaks in public schools, which account for 77% of all schools in Argentina, because we did not have data on winter school breaks from private schools, which might follow a different school calendar (*23*). We could assume, however, that bias resulting from the incomplete representativeness of the SNVS data and from the school calendar data would move our results toward the null hypothesis.

Another potential limitation of school-closure studies that rely on surveillance data are that observed reductions in disease might be caused by changes in health care–seeking behavior associated with the break and might not represent actual disease reductions. Families might be less likely to seek care during holidays because of travel or other reasons. This limitation is particularly relevant because we analyzed numbers of ILI cases, not ILI rates, as a percentage of all medical visits, as is commonly done to monitor the spread of influenza in the United States (*24*). However, we found that the greatest reductions in ILI were observed in the first week after school reopening, suggesting that the observed decreases in ILI represent true decreases in ILI rather than changes in health care–seeking behavior.

Despite these limitations, a strength of our study is that because the school calendars were set at the beginning of each school year, the timing of winter school breaks was independent of the timing of ILI activity. Furthermore, the dates for winter holidays varied by province and by year, thus allowing for greater differences between a given province’s winter school break dates and the province’s respective epidemiologic curve. This difference provides our analysis with another source of variation in the explanatory, independent of ILI incidence, resulting in more robust results.

Because of the inherent limitations of ecologic studies, it would be ideal to perform prospective field studies to actually assess the effectiveness of school closures (*25*). Before the emergence of pandemic influenza (H1N1) virus in 2009, most field studies that looked at school closure were in the context of a reactive school closure (i.e., schools were closed because of substantial disease or absenteeism and/or the study group lacked a comparison group), making it difficult to determine the effectiveness of the school closure on disease circulation (*7*,*26*). A study in Israel took advantage of a teachers strike to study the effect of school closure on respiratory diseases and found evidence of an effect on incidence (*14*). A study in Canada found evidence of significant effect of school closures and changes in the weather on the incidence of transmission of pandemic influenza (H1N1) virus (*27*). Another study in a large metropolitan area in the United States took advantage of a natural experiment, in which 1 school district closed its schools for 10 days during the 2009 influenza pandemic, while most schools in a neighboring school district did not close. In that study, the authors found that school closure was associated with fewer self-reported cases of acute respiratory illness and fewer visits to the emergency department for ILI-associated conditions (*28*).

Although school closures might be useful as a mitigation measure during influenza seasons, many additional questions about school closure remain and deserve attention, such as the duration of the closure and the timing with respect to the influenza season. Moreover, closure should probably be accompanied by instructions to not congregate elsewhere. Questions remain about the incidence rate needed to trigger a closure; the social behavior of children when not in school; and the effect of school closure on children, their families, and society. For example, a recent study in Argentina found that the cost of school closure falls disproportionately on the poor (*29*).

Although the effect of winter school breaks was found to be modest, the reduction in disease transmission associated with school closure might slow spread of disease and lessen the effect on hospitals and other health care providers, thus affording extra time to triage limited resources. These factors might be especially crucial when intensive care capacity or antiviral availability are limited, such as in the early stages of a pandemic. Our findings provide additional data for policy makers and public health officials to use when considering such measures to control pandemic influenza.

Technical Appendix Poisson regression model.
