# Supplementary material for: Effect of Winter School Breaks on Influenza-like Illness, Argentina, 2005–2008
Source: Emerg Infect Dis. 2013 Jun;19(6):938–44. doi: 10.3201/eid1906.120916 (PMC3713818; doi:10.3201/eid1906.120916)
Supplement: Technical Appendix — Poisson regression model. [file 12-0916-Techapp-s1.pdf]

Article DOI: http://dx.doi.org/10.3201/eid1906.120916

Effect of Winter School Breaks on Influenza-like Illness, Argentina, 2005–2008

Technical Appendix

The following Poisson regression model was estimated for each age group separately:

$$ILI_{p,t} = \alpha_p \cdot exp\ \ \beta_0 + \beta_1 ILI_{p,t-1} + \beta_2 t + \beta_3 S_t$$
$$+ \beta_4 Y_t + \beta_5 R_p + \beta_6 S_t \cdot Y_t + \beta_7 S_t \cdot R_p + \beta_8 W_{p,t} + \beta_9 U_{p,t}$$

In this equation, each β is a vector of coefficients, *p* and *t* are sub-indices for province and week number, respectively, and

- $ILI_{p,t}$ is the number of ILI-related medical visits in province *p* and week *t*,
- α_p is the population size of the corresponding age group in province *p*, as reported in the 2001 Argentinean population census,
- $ILI_{p,t-1}$ is a lagged term for the number of ILI-related medical visits in province *p* and week *t–1*,
- $t = \ t \quad t^2$, are linear and quadratic time trends, where *t* is the week number from Jan 1, 2005 to Dec 31, 2008,
- $S_t = \ \sin\frac{2t\pi}{52} \quad \cos\frac{2t\pi}{52} \quad \sin\frac{4t\pi}{52} \quad \cos\frac{4t\pi}{52}$ is a vector of sinusoidal terms to account for seasonal patterns in the number of ILI-related visits,
- $Y_t = \ y_{2005}\ y_{2006}\ y_{2007}\ y_{2008}$ is a vector of dummy variables for each year,
- $R_p = \ r_1 \quad r_2 \quad r_3\ r_4 \quad r_5 \quad r_6$ is a vector of dummy variables for the geographic region where each province is located,
- $W_{p,t} = \ w_1 \quad w_2$ is a vector of dummy variables for the first and second weeks of the winter school breaks in each province and year, and

- $U_{p,t} = \begin{bmatrix} u_1 & \cdots & u_{10} \end{bmatrix}$ are dummy variables for the five weeks before and the five weeks after the winter school breaks in each province and year.

The interactions between the sinusoidal effects and the years (S_t, Y_t) and regions (S_t, R_p) allow for differences in the seasonality of ILI visits across geographic regions and within each year.

We report the coefficients of the W_{pt} and U_{pt} as incidence rate ratios (IRRs), which can then be interpreted as the difference of the incidence of ILI visits during those weeks from their estimated seasonal patterns. That is, if an IRR is <1 for a particular week, this would indicate that ILI activity during that week was lower than would have been expected given the estimated seasonal trends of ILI visits.
